# Pharmacological therapy in patients diagnosed with Peyronie's disease

**Published:** 2012-06-18

**Authors:** AA Halal, P Geavlete, E Ceban

**Affiliations:** * MEDAS Medical Center, Bucharest; ** “Sf. Ioan” Clinical Emergency Hospital, Department of Urology, Bucharest; *** "Nicolae Testemişanu" USMF, Chișinău, Republic of Moldova

**Keywords:** Peyronie disease, verapamil, vitamin E, colchicine

## Abstract

Peyronie's disease is still a therapeutic dilemma for the urologist. Although medical treatment includes multiple versions, few therapeutic agents had significant effects. The combination between oral therapy and intralesional agents can improve the quality of life of the patients with Peyronie's disease.

## Introduction

Peyronie's disease is a disorder manifested by the abnormal penis curvature during erection, due to the development of fibrous nodules in the albuginée, first described in 1743 by Francois Gigot de La Peyronie [**1**], associated or not with pain and the inability to achieve a satisfactory sexual intercourse. The best therapy of Peyronie's disease remains controversial, in part because the etiology and natural history of the disease are not sufficiently known, and the treatment aims not to the disease itself but its effects (penile curvature and erectile dysfunction). The disease was associated with other systemic conditions such as hypertension and diabetes, Dupuytren's disease and Ledderhose’s disease.

While severe deformities and erectile dysfunctionare best treated surgically, for light and medium curvatures, pain and slow disease progression is preferred to a conservative approach by oral and intralesional therapy [**2,3**].

## Methods

**Fig. 1:**
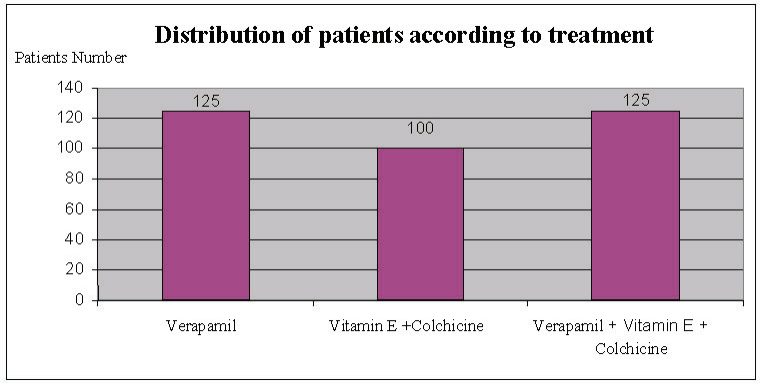
Therapeutical study groups

During 2004-2012, 350 patients diagnosed with Peyronie's disease, were treated as it follows: group I consisted of 125 patients treated with intralesional injections of verapamil; second group of 100 patients were treated with Vitamin E and Colchicine; third group consisted of 125 patients treated with verapamil, vitamin E and Colchicine (**Fig. 1**). The age of patients was between 19 and 72 years.

The group of patients underwent a comprehensive diagnostic protocol that included medical history, clinical examination, self-photography, penile Doppler ultrasound in patients with associated erectile dysfunction. Following investigations and clinical examination have determined the way the disease started, the place of the injury, size and number of calcifications present, presence of pain, degree of penile deviation, associated erectile dysfunction and the presence of other affections. 

Treatment protocol included 10 mg Verapamil, one injection per week for a period of 12 to 15 weeks, 200 mg vitamin E twice daily in combination with Colchicine 1mg twice a day for a period of 3 to 6 months and the association of the three therapeutic agents for a period of 3 months.

Drug therapy was administered as it follows: local anesthesia with lidocaine after local aseptic Betadine; infiltration is carried out in several places in the plaque to ensure the uniform distribution of the injected drug and then the place must be compressed for a few minutes in order to reduce local bruising. The injection was done with care to avoid the damage of the neurovascular bundle and the penetration of the drug in the corpus cavernosum.

## Results

**Fig. 2:**
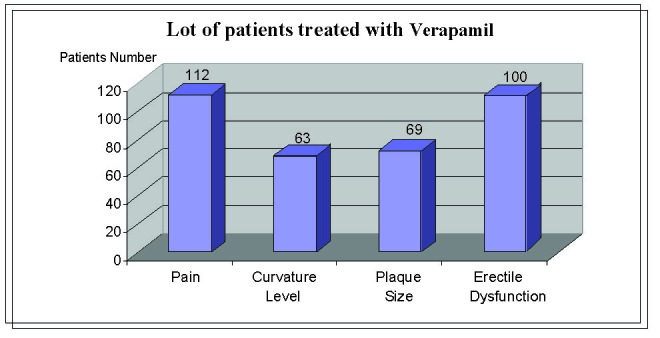
Results in patients treated with verapamil

In the first group (125 patients) treated with verapamil, followed by a period of 12 to 15 weeks, we found the following results (**Fig. 2**):

- Pain resolved completely or partially in 90% of the patients (112 patients)

- Curvature level decreased by 10 – 30 in 50% of the patients (63 patients), while for the rest there was no change in the deviation

- Plaque size decreased to about 55% of the patients (69 patients), while the rest remained unchanged

- improved erectile dysfunction in 80% of the patients (100 patients)

**Fig. 3:**
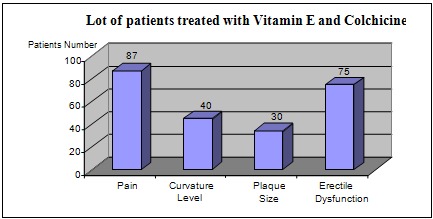
Results in patients treated with vitamin E and colchicine

In the group of 100 patients treated with vitamin E and colchicine, we have noticed the following (Fig. 3):

- pain resolved completely or partially in 87% of the patients (87 patients)

- curvature level decreased by 10 – 20 in 40% of the patients (40 patients), while for the rest there was no change in the deviation

- plaque size decreased to about 30% of the patients (30 patients), while the rest remained unchanged

- improved erectile dysfunction in 75% of the patients (75 patients)

**Fig. 4:**
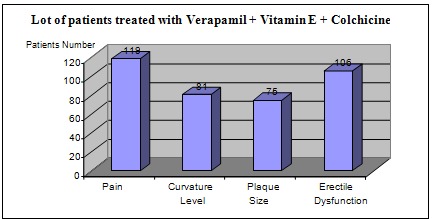
Results in patients treated with verapamil, vitamin E and colchicine

For the group of 125 patients treated with verapamil in combination with Vitamin E and Colchicine, the results were the following (**Fig. 4**):

- pain resolved completely or partially in 95% of the patients (119 patients)

- curvature level decreased by 20 – 45 in 65% of the patients (81 patients), while for the rest there was no change in the deviation

- plaque size decreased to about 60% of the patients (75 patients), while the rest remained unchanged

- improved erectile dysfunction in approx. 85% of the patients (106 patients)

**Table 1:** Results in all three cases (treatment with verapamil; vitamin E and colchicine; verapamil, vitamin E and colchicine)

	Verapamil	Vitamin E + Colchicine	Verapamil + Vitamin E + Colchicine
Total patients	125 patients	100 patients	125 patients
Pain – resolved	90 % - 112 patients	87 % - 87 patients	95% - 119 patients
Curvature Level – decreased	50 % - 63 patients 10 º -30º	40 % - 40 patients 10 º -20º	65% - 81 patients 20 º -45 º
Plaque Size – decreased	55 % - 69 patients	30 % - 30 patients	60% - 75 patients
Erectile Dysfunction – improved	80 % - 100 patients	75 % - 75 patients	85% - 106 patients

## Discussions

Candidates for medical treatment of Peyronie's disease are patients with an early stage disease, those with progressive deformity and unstable plaque and those with painful erections.

Relatively recent studies have shown that calcium channel blockers (especially verapamil) are effective in the treatment of Peyronie's disease when administered intralesional (or perilesional). When administered orally, verapamil is effective for cardiologic use. Verapamil has the following benefic effects in this condition: increase in extracellular collagenase activity, decreased metabolic activity of fibroblasts, extracellular collagen deposit reduction [**4-6**]. Several regimens such as the following can be used: 10 mg per week for 10 weeks or 10 mg every two weeks – 10 doses. Other schemes require 2-3 cures repeated at various intervals, using the same dose (10 mg every week or at 2 weeks). The following were reported as side effects: bruises, hematomas, abnormal sensitivity (temporary). There were no reported episodes of hypotension, cardiac arrhythmias or local infection after intralesional administration of verapamil in literature. In assessing the therapeutic efficacy, the following were evaluated: plaque size, penile curvature level, severity of pain in erection. In 1996, Levine studied 38 patients treated with verapamil 10 mg for 2 weeks (12 infiltrations). He noted a rapid reduction in pain in 97% of the patients, subjective improved of curvature in 76% of the patients, reduced plaque thickness in 86% of the patients, improved rigidity distal in 93% and improved erectile performance in 72% of the patients. Plaque volume increased in 43% of the patients, but gradually decreased after 6-18 months. In 65% of the patients with disease duration less than one year, the curvature decreased by 21 on average and 44% of those with disease duration longer than one year, the curvature decreased by 23. Complications or side effects were minimal, with no infection, hypotension or arrhythmias [**7-9**].

Vitamin E has been much used in the treatment of Peyronie's disease for its antioxidant effect to prevent fibrosis. Although changes of curvature and plaque are not significant [**10**], because it is a cheap drug, and it has an apparent effect of pain reduction and reduced side effects, vitamin E can be considered in the treatment of Peyronie's disease. Yet, early studies showed reduced curvature in 78% of the patients and reduction in size calcifications in 91% of the patients [**11-13**].

Colchicine was recently recognized as having a therapeutic potential in Peyronie's disease. It works by blocking the path of arachidonic acid lipoxygenase, thus preventing leukotriene formation by reducing inflammation and chemotaxis and interferes with procollagen transcellular migration, reducing the formation of procollagen and enhancing the production of collagenase [**14**]. Therefore, it has antifibrotic, antimitotic and anti-inflammatory effects. Akkus et al. [**15**] published the ﬁrst pilot study in which they administered oral colchicine for 3–5 months to a group of 24 patients with Peyronie’s disease, with encouraging results.

## Conclusions

Treatment with intralesional Verapamil proved to be extremely useful in the early stages of Peyronie's disease with positive effects on pain, curvature and plaque size. However, the association of Verapamil with Vitamin E and Colchicine showed better pain control while reducing penile curvature, dimensions of calcification and the degree of erectile dysfunction, thus improving the quality of life.
